# Supplementary material for: Anxiety and depression symptoms, the recovery from symptoms, and loneliness before and after the COVID-19 outbreak among the general population: Findings from a Dutch population-based longitudinal study
Source: PLoS One. 2021 Jan 7;16(1):e0245057. doi: 10.1371/journal.pone.0245057 (PMC7790276; doi:10.1371/journal.pone.0245057)
Supplement: S1 Appendix — Cross-tabulation loneliness classes at T1 and T4. (DOCX) [file pone.0245057.s001.docx]

S1 Appendix. Table S1 Cross-tabulation loneliness classes at T1 and T4

|  | class 1 | class 2 | class 3 | class 4 |  |
| --- | --- | --- | --- | --- | --- |
|  | n | n | n | n | Total |
| Low loneliness (class 1) | 2,559 | 494 | 11 | 31 | 3,095 |
| Emotional loneliness (class 2) | 248 | 451 | 44 | 11 | 754 |
| Social and emotional loneliness (class 3) | 23 | 56 | 75 | 14 | 168 |
| Social loneliness (class 4) | 32 | 12 | 12 | 11 | 67 |
| Total | 2,862 | 1,013 | 142 | 67 | 4,084 |

Due to weighting, numbers may slightly differ between Tables
