# Supplementary material for: Anxiety and depression symptoms, the recovery from symptoms, and loneliness before and after the COVID-19 outbreak among the general population: Findings from a Dutch population-based longitudinal study
Source: PLoS One. 2021 Jan 7;16(1):e0245057. doi: 10.1371/journal.pone.0245057 (PMC7790276; doi:10.1371/journal.pone.0245057)
Supplement: S2 Appendix — Recovery in the months following at T2 and T3. (DOCX) [file pone.0245057.s002.docx]

S2 Appendix. Table S2 Recovery in the months following at T2 and T3

|  | High anxiety and depression symptoms at T3/T4 | | | | | | |
| --- | --- | --- | --- | --- | --- | --- | --- |
|  |  |  |  | split-half groups^1^ | | | |
|  | Total group | |  | Subgroup A1 | | Subgroup B1 | |
|  | n | % |  | n | % | N | % |
| Anxiety and depression symptoms at T2 | | | | | | | |
| - remission at T3 | 43 | 16.9 |  | 20 | 16.4 | 23 | 17.0 |
| - improved at T3 | 29 | 11.1 |  | 16 | 13.1 | 12 | 8.9 |
| - unchanged at T3 | 132 | 51.1 |  | 59 | 48.4 | 73 | 54.1 |
| - worsened at T3 | 54 | 20.9 |  | 27 | 22.1 | 27 | 20.0 |
|  |  |  |  |  | | | |
|  | Total group | |  | Subgroup A2 | | Subgroup B2 | |
|  | N | % |  | N | % | n | % |
| Anxiety and depression symptoms at T3 | | | | | | | |
| - remission at T4 | 42 | 17.7 |  | 20 | 18.9 | 22 | 16.7 |
| - improved at T4 | 30 | 12.8 |  | 15 | 14.2 | 16 | 12.1 |
| - unchanged at T4 | 131 | 55.2 |  | 52 | 49.1 | 79 | 59.8 |
| - worsened at T4 | 34 | 14.3 |  | 19 | 17.9 | 15 | 11.4 |

^1^The total study sample (N=4084) was randomly split in two exclusive subgroups (subgroup A=2038, subgroup B=2046, due to weighting the distribution is not exactly 50-50%). For all four subgroups recovery was computed like the computations of recovery at T3 and T4 among the total group with high symptom levels at T2 and T3, using the M's and SD's of the four subgroups.

A1 versus B2: χ2=6.04, df=3, p=0.11. A2 versus B1: χ^2^=2.00, df=3, p=0.572
