# Supplementary material for: Anxiety and depression symptoms, the recovery from symptoms, and loneliness before and after the COVID-19 outbreak among the general population: Findings from a Dutch population-based longitudinal study
Source: PLoS One. 2021 Jan 7;16(1):e0245057. doi: 10.1371/journal.pone.0245057 (PMC7790276; doi:10.1371/journal.pone.0245057)
Supplement: S3 Appendix — Results LCA loneliness. (DOCX) [file pone.0245057.s003.docx]

S3 Appendix. Table S3 Results LCA loneliness

| Model | Classes | LL | BIC(LL) | SABIC(LL) | BVR ≥ 3.84 | N^par^ | Class. Err. | Entropy R | N |  |  |  |  |  |  |
| --- | --- | --- | --- | --- | --- | --- | --- | --- | --- | --- | --- | --- | --- | --- | --- |
| Pre-outbreak (T1) | | | | | | | | | | | | | | | |
| Model1 | 1-Class | -9,635.94 | 19,321.76 | 19302.70 | yes | 6.00 | 0.00 | 1.00 | 4,084 |  |  |  |  |  |  |
| Model2 | 2-Classes | -7,548.89 | 15,205.88 | 15164.57 | yes | 13.00 | 0.02 | 0.93 | 3,077 | 1,007 |  |  |  |  |  |
| Model3 | 3-Classes | -7,236.58 | 14,639.45 | 14575.90 | yes | 20.00 | 0.03 | 0.93 | 3,103 | 747 | 234 |  |  |  |  |
| ***Model4*** | ***4-Classes*** | ***-7,142.17*** | ***14,508.84*** | ***14423.04*** | ***no*** | ***27.00*** | ***0.04*** | ***0.93*** | ***3,095*** | ***753*** | ***168*** | ***68*** |  |  |  |
| Model5 | 5-Classes | -7,134.48 | 14,551.66 | 14443.62 | no | 34.00 | 0.13 | 0.81 | 2,899 | 703 | 204 | 211 | 67 |  |  |
| Model6 | 6-Classes | -7,128.93 | 14,598.77 | 14468.49 | no | 41.00 | 0.12 | 0.84 | 2,744 | 614 | 421 | 208 | 42 | 54 |  |
| Model7 | 7-Classes | -7,124.22 | 14,647.54 | 14495.02 | no | 48.00 | 0.11 | 0.85 | 2,744 | 614 | 464 | 162 | 59 | 25 | 16 |
| Post-outbreak (T4) | | | | | | | | | | | | | | | |
| Model1 | 1-Class | -9,701.02 | 19,451.93 | 19432.87 | yes | 6.00 | 0.00 | 1.00 | 4,084 |  |  |  |  |  |  |
| Model2 | 2-Classes | -8,208.28 | 16,524.66 | 16483.35 | yes | 13.00 | 0.04 | 0.90 | 2,842 | 1,242 |  |  |  |  |  |
| Model3 | 3-Classes | -7,741.32 | 15,648.94 | 15585.39 | yes | 20.00 | 0.04 | 0.92 | 2,862 | 1,003 | 219 |  |  |  |  |
| ***Model4*** | ***4-Classes*** | ***-7,669.36*** | ***15,563.21*** | ***15477.42*** | ***no*** | ***27.00*** | ***0.05*** | ***0.91*** | ***2,862*** | ***1,014*** | ***142*** | ***67*** |  |  |  |
| Model5 | 5-Classes | -7,654.07 | 15,590.84 | 15482.81 | no | 34.00 | 0.06 | 0.88 | 2,862 | 897 | 200 | 71 | 55 |  |  |
| Model6 | 6-Classes | -7,648.73 | 15,638.36 | 15508.08 | no | 41.00 | 0.06 | 0.88 | 2,861 | 889 | 120 | 99 | 58 | 57 |  |
| Model7 | 7-Classes | -7,644.82 | 15,688.74 | 15536.22 | no | 48.00 | 0.12 | 0.85 | 2,192 | 1229 | 404 | 121 | 60 | 60 | 19 |

The best fitting model is presented in bold italics. LL=log likelihood. BIC=Bayesian information criterion (BIC). SABIC=sample size adjusted BIC. BVR=Bi-variate residuals. Class. Err.=Classification error. N^par^=number of estimated parameters. N=4084.
