# Supplementary material for: Anxiety and depression symptoms, the recovery from symptoms, and loneliness before and after the COVID-19 outbreak among the general population: Findings from a Dutch population-based longitudinal study
Source: PLoS One. 2021 Jan 7;16(1):e0245057. doi: 10.1371/journal.pone.0245057 (PMC7790276; doi:10.1371/journal.pone.0245057)
Supplement: S4 Appendix — Latent class profiles of loneliness at T1 and T4. (DOCX) [file pone.0245057.s004.docx]

S4 Appendix. Table S4 Latent class profiles of loneliness at T1 and T4

|  | | I have a sense of emptiness around me | there are enough people I can count on in case of a misfortune | I know a lot of people that I can fully rely on | there are enough people to whom I feel closely connected | I miss having people around me | I often feel deserted |  |
| --- | --- | --- | --- | --- | --- | --- | --- | --- |
|  | N | Probability of endorsing loneliness items (estimated values) | | | | | | Label |
| Class 1 | 3,095/2,862 | 0.03/0.03 | 0.01/0.00 | 0.03/0.02 | 0.01/0.01 | 0.07/0.23 | 0.02/0.02 | low loneliness |
| Class 2 | 753/1014 | 0.86/0.89 | 0.04/0.02 | 0.07/0.04 | 0.06/0.03 | 0.91/0.94 | 0.58/0.39 | emotional loneliness |
| Class 3 | 168/142 | 0.96/0.92 | 0.53/0.63 | 0.91/0.85 | 0.69/0.70 | 0.93/0.90 | 0.81/0.83 | social and emotional loneliness |
| Class 4 | 68/67 | 0.20/0.12 | 0.70/0.63 | 0.87/0.96 | 0.84/0.70 | 0.08/0.11 | 0.09/0.16 | social loneliness |

Numbers before the slash (/) refer to the results of LCA on T1 data, and numbers after the slash refer to results on T4 data.
