# Supplementary material for: Anxiety and depression symptoms, the recovery from symptoms, and loneliness before and after the COVID-19 outbreak among the general population: Findings from a Dutch population-based longitudinal study
Source: PLoS One. 2021 Jan 7;16(1):e0245057. doi: 10.1371/journal.pone.0245057 (PMC7790276; doi:10.1371/journal.pone.0245057)
Supplement: Attachment — Submitted filename: Manuscript with comments.pdf [file pone.0245057.s005.pdf]

PLOS ONE

Anxiety and depression symptoms, the recovery from symptoms and loneliness before and after the COVID-19 outbreak among the general population. Findings from a Dutch population-based longitudinal study

--Manuscript Draft--

**Manuscript Number:**	PONE-D-20-31580
**Article Type:**	Research Article
**Full Title:**	Anxiety and depression symptoms, the recovery from symptoms and loneliness before and after the COVID-19 outbreak among the general population. Findings from a Dutch population-based longitudinal study
**Short Title:**	Anxiety, depression and loneliness before and after COVID-19 outbreak
**Corresponding Author:**	Peter G van der Velden, Ph.D.<br>CentERdata<br>Tilburg, NETHERLANDS
**Keywords:**	Covid-19; Anxiety and depression symptoms; Emotional loneliness; Social loneliness; Prospective; Prevalence; Reliable Change Index (RCI); LISS; VICTIMS-study; General population
**Abstract:**	Objectives. The aim of the present longitudinal study was to gain insight in the effects of the COVID-19 pandemic on the mental health and loneliness in the general population. More specifically, the study focused on prevalence of anxiety and depression symptoms , the extent to which individuals with existing symptoms recovered or not, the prevalence of subtypes of loneliness, and the extent to which loneliness before and during this pandemic was associated with anxiety and depression symptoms.<br>Methods. Data was extracted from the longitudinal LISS panel, based on a probability sample of the Dutch population, with assessments on loneliness in October 2019 (T1) and June 2020 (T4), and anxiety and depression symptoms in November 2019 (T2), March 2020 (T3) and June 2020 (T4; N total =4,084). Loneliness was examined with the De Jong Gierveld Loneliness Scale and anxiety and depression symptoms with the Mental Health Inventory (MHI-5).<br>Results. Repeated multivariate logistic regression analyses (MLRA) showed a lower prevalence of anxiety and depression symptoms after the outbreak (T4=15.3%) than before (T2=16.8%) and during the COVID-19 outbreak (T3=17.2%). According to the Reliable Change Index, the distribution of recovery categories (remission, improvement, unchanged and worsening symptoms) after the outbreak did not differ from the distribution of these categories before the outbreak. Repeated MLRA revealed that the prevalence of emotional loneliness increased after the outbreak (T1=18.4%, T4=24.8%). Among individuals who were not lonely before and after the outbreak the prevalence of symptoms decreased (T2=7.0%, T4=4.4%) and, likewise, among those who were not lonely anymore after the outbreak (T2=21.5%, T4=14.5%). However, the prevalence of symptoms increased among those who became lonely during the pandemic (T2=17.9%, T4=26.3%).<br>Conclusions. Findings suggest that this pandemic did not negatively affect the prevalence of anxiety and depression symptoms nor the normal recovery of symptoms among the general population during the first four months, but that emotional loneliness increased.
**Order of Authors:**	Peter G van der Velden, Ph.D.
	Philip Hyland
	Carlo Contino
	Hans-Martin von Gaudecker
	Ruud Muffels
	Marcel Das
**Additional Information:**	

Powered by Editorial Manager® and ProduXion Manager® from Aries Systems Corporation

Question	Response
**Financial Disclosure**<br><br>Enter a financial disclosure statement that describes the sources of funding for the work included in this submission. Review the submission guidelines for detailed requirements. View published research articles from *PLOS ONE* for specific examples.<br><br>This statement is required for submission and **will appear in the published article** if the submission is accepted. Please make sure it is accurate.<br><br>**Unfunded studies**<br>Enter: *The author(s) received no specific funding for this work.*<br><br>**Funded studies**<br>Enter a statement with the following details:<br>• Initials of the authors who received each award<br>• Grant numbers awarded to each author<br>• The full name of each funder<br>• URL of each funder website<br>• Did the sponsors or funders play any role in the study design, data collection and analysis, decision to publish, or preparation of the manuscript?<br>• **NO** - Include this sentence at the end of your statement: *The funders had no role in study design, data collection and analysis, decision to publish, or preparation of the manuscript.*<br>• **YES** - Specify the role(s) played.<br><br>* typeset	This work was supported by Fonds Slachtofferhulp, The Haque, The Netherlands (50006/VICTIMS). One of the authors (CC) is deputy director at Fonds Slachtofferhulp.<br><br>The funder had no further role in study design, data collection and analysis, decision to publish, or preparation of the manuscript.
**Competing Interests**<br><br>Use the instructions below to enter a competing interest statement for this submission. On behalf of all authors, disclose any competing interests that could be perceived to bias this work—acknowledging all financial support and any other relevant financial or non-financial competing interests.<br><br>This statement **will appear in the published article** if the submission is	The authors have declared that no competing interests exist.

accepted. Please make sure it is accurate. View published research articles from *PLOS ONE* for specific examples.<br><br>**NO authors have competing interests**<br><br>Enter: *The authors have declared that no competing interests exist.*<br><br>**Authors with competing interests**<br><br>Enter competing interest details beginning with this statement:<br><br>*I have read the journal's policy and the authors of this manuscript have the following competing interests: [insert competing interests here]*<br><br>* typeset	
**Ethics Statement**<br><br>Enter an ethics statement for this submission. This statement is required if the study involved:<br><br>• Human participants<br>• Human specimens or tissue<br>• Vertebrate animals or cephalopods<br>• Vertebrate embryos or tissues<br>• Field research<br><br>Write "N/A" if the submission does not require an ethics statement.<br><br>General guidance is provided below. Consult the submission guidelines for detailed instructions. **Make sure that all information entered here is included in the Methods section of the manuscript.**	According to the Dutch Medical Research Involving Human Subjects Act (WMO) the present study did not require approval from a Medical Ethical Testing Committee (METC). Nevertheless, the longitudinal Social Integration and Leisure module and Health module (as part of the Longitudinal Core Study in LISS, starting in 2007) were evaluated and approved by the Board of Overseers, an Internal Review Board (IRB) until 2014. The VICTIMS survey (starting in 2018) was approved by an Institutional Review Board, consisting of external and internal reviewers of CentERdata. The ‘Effects of the Outbreak of Covid-19’ survey was conducted by the Institute for Applied Microeconomics of Universität Bonn in Germany, and in Germany no medical ethical approval is required for this type of research. The ‘Effects of the Outbreak of COVID-19’ survey was evaluated and approved by internal reviewers of CentERdata not involved in designing this study, and used the same questions on mental health and loneliness as the surveys approved by the IRB. In accordance with the General Data Protection Regulation (GDPR), participants gave explicit consent for the use of the collected data for scientific and policy relevant research.

Powered by Editorial Manager® and ProduXion Manager® from Aries Systems Corporation

**Format for specific study types**<br><br>**Human Subject Research (involving human participants and/or tissue)**<br>• Give the name of the institutional review board or ethics committee that approved the study<br>• Include the approval number and/or a statement indicating approval of this research<br>• Indicate the form of consent obtained (written/oral) or the reason that consent was not obtained (e.g. the data were analyzed anonymously)<br><br>**Animal Research (involving vertebrate animals, embryos or tissues)**<br>• Provide the name of the Institutional Animal Care and Use Committee (IACUC) or other relevant ethics board that reviewed the study protocol, and indicate whether they approved this research or granted a formal waiver of ethical approval<br>• Include an approval number if one was obtained<br>• If the study involved *non-human primates*, add *additional details* about animal welfare and steps taken to ameliorate suffering<br>• If anesthesia, euthanasia, or any kind of animal sacrifice is part of the study, include briefly which substances and/or methods were applied<br><br>**Field Research**<br><br>Include the following details if this study involves the collection of plant, animal, or other materials from a natural setting:<br>• Field permit number<br>• Name of the institution or relevant body that granted permission	
**Data Availability**<br><br>Authors are required to make all data underlying the findings described fully available, without restriction, and from the time of publication. PLOS allows rare exceptions to address legal and ethical concerns. See the PLOS Data Policy and FAQ for detailed information.	Yes - all data are fully available without restriction

A Data Availability Statement describing where the data can be found is required at submission. Your answers to this question constitute the Data Availability Statement and **will be published in the article**, if accepted.<br><br>**Important:** Stating 'data available on request from the author' is not sufficient. If your data are only available upon request, select 'No' for the first question and explain your exceptional situation in the text box.<br><br>Do the authors confirm that all data underlying the findings described in their manuscript are fully available without restriction?	
**Describe where the data may be found in full sentences. If you are copying our sample text, replace any instances of XXX with the appropriate details.**<br><br>• If the data are **held or will be held in a public repository**, include URLs, accession numbers or DOIs. If this information will only be available after acceptance, indicate this by ticking the box below. For example: *All XXX files are available from the XXX database (accession number(s) XXX, XXX.).*<br>• If the data are all contained **within the manuscript and/or Supporting Information files**, enter the following: *All relevant data are within the manuscript and its Supporting Information files.*<br>• If neither of these applies but you are able to provide **details of access elsewhere**, with or without limitations, please do so. For example:<br><br>*Data cannot be shared publicly because of [XXX]. Data are available from the XXX Institutional Data Access / Ethics Committee (contact via XXX) for researchers who meet the criteria for access to confidential data.*<br><br>*The data underlying the results presented in the study are available from (include the name of the third party*	The study was conducted using the Dutch Longitudinal Internet studies for the Social Sciences (LISS) panel [27]. The LISS panel started in 2007 and is based on a large traditional probability sample drawn from the Dutch population. The Dutch Research Counsel (NWO) funded the set-up of LISS. Panel members receive an incentive of €15 per hour for their participation and those who do not have a computer and/or Internet access are provided with the necessary equipment at home. Further information about all conducted surveys and regulations for free access to the data, as well as the data files can be found at www.lissdata.nl (in English). The LISS panel has received the international Data Seal of Approval (see https://www.datasealofapproval.org/ en/). All data of studies conducted with the LISS panel are anonymized. Data of surveys conducted in 2020 will be added to the open access data archive soon.

Powered by Editorial Manager® and ProduXion Manager® from Aries Systems Corporation

and contact information or URL).<br>• This text is appropriate if the data are owned by a third party and authors do not have permission to share the data.<br><br>* typeset	
Additional data availability information:	Tick here if the URLs/accession numbers/DOIs will be available only after acceptance of the manuscript for publication so that we can ensure their inclusion before publication.

Manuscript

Click here to access/download;Manuscript;NL before and after the COVID manuscript 7-10-2020.docx

Anxiety and depression symptoms, the recovery from symptoms and loneliness before and after the COVID-19 outbreak among the general population. Findings from a Dutch population-based longitudinal study

Anxiety, depression and loneliness before and after COVID-19 outbreak

Peter G. van der Velden[1,2], Philip Hyland[3] , Carlo Contino[4], Hans-Martin von Gaudecker[5] , Ruud Muffels[6], and Marcel Das[1,7]

[1] CentERdata, Tilburg, The Netherlands

[2] Tilburg University's Network on Health and Labor (NETHLAB),Tilburg, The Netherlands

[3] Department of Psychology, Maynooth University, Kildare, Ireland.

[4] Fonds Slachtofferhulp, The Hague, The Netherlands

[5] Institute for Applied Microeconomics, Universität Bonn, Germany

[6] Tilburg School of Social and Behavioral Sciences, and Tranzo (Scientific center for care and welfare), Tilburg University, Tilburg, the Netherlands

[7] Tilburg School of Economics and Management, Tilburg University, Tilburg, The Netherlands

* Corresponding author

Email: pg.vandervelden@tilburguniversity.edu

ABSTRACT

Objectives. The aim of the present longitudinal study was to gain insight in the effects of the COVID-19 pandemic on the mental health and loneliness in the general population. More specifically, the study focused on prevalence of anxiety and depression symptoms, the extent to which individuals with existing symptoms recovered or not, the prevalence of subtypes of loneliness, and the extent to which loneliness before and during this pandemic was associated with anxiety and depression symptoms.

Methods. Data was extracted from the longitudinal LISS panel, based on a probability sample of the Dutch population, with assessments on loneliness in October 2019 (T1) and June 2020 (T4), and anxiety and depression symptoms in November 2019 (T2), March 2020 (T3) and June 2020 (T4; N^{total}=4,084). Loneliness was examined with the De Jong Gierveld Loneliness Scale and anxiety and depression symptoms with the Mental Health Inventory (MHI-5).

Results. Repeated multivariate logistic regression analyses (MLRA) showed a lower prevalence of anxiety and depression symptoms after the outbreak (T4=15.3%) than before (T2=16.8%) and during the COVID-19 outbreak (T3=17.2%). According to the Reliable Change Index, the distribution of recovery categories (remission, improvement, unchanged and worsening symptoms) after the outbreak did not differ from the distribution of these categories before the outbreak. Repeated MLRA revealed that the prevalence of emotional loneliness increased after the outbreak (T1=18.4%, T4=24.8%). Among individuals who were not lonely before and after the outbreak the prevalence of symptoms decreased (T2=7.0%, T4=4.4%) and, likewise, among those who were not lonely anymore after the outbreak (T2=21.5%, T4=14.5%). However, the

prevalence of symptoms increased among those who became lonely during the pandemic (T2=17.9%, T4=26.3%).

Conclusions. Findings suggest that this pandemic did not negatively affect the prevalence of anxiety and depression symptoms nor the normal recovery of symptoms among the general population during the first four months, but that emotional loneliness increased.

Keywords

COVID-19; Anxiety and depression symptoms; Emotional loneliness; Social loneliness; Prospective; Prevalence; Reliable Change Index (RCI); LISS; VICTIMS-study; General population.

Introduction

The COVID-19 pandemic negatively affects countries on a micro-, meso- and macro- level. At the micro-level it varies from the threat of people becoming infected or when infected, becoming very ill or even die, loss of significant others, unemployment, and social isolation because of social distancing rules, to reduced health care use among non-COVID-19 patients [1]. At meso and macro-levels it leads to understaffing and reduced revenues of firms, increasing governmental deficits, increasing economic insecurity, changing demand patterns of consumers [2], and collapsing growth levels in the global economy [3,4]. A key question is to what extent this pandemic and the uncertainties about the future will negatively affect the mental health of the general population.

The number of empirical studies aimed at answering this question is increasing very rapidly, creating the risk recently published but important studies are overlooked. Nevertheless, current studies suggest that this pandemic increases the risk of mental health problems among the general population [5,6]. However, many of these studies used cross-sectional study designs and are based on convenience samples instead of probability samples of the general population. A recent review by Xiong et al. [6] showed that all 19 identified and included COVID-19 studies used cross-sectional study designs and that 18 out of 19 studies used (potentially biased) convenience samples. These convenience samples used, for instance, snowball techniques and messages on social media as strategies to recruit respondents.

To the best of our knowledge, to date five prospective population-based studies with pre- and post-COVID-19 data have been published using probability samples of the general population to assess the effects of the COVID-19 pandemic on the mental health of the general population. Twenge and Joiner [7] assessed the effects in the United States (US) by comparing

anxiety and depression symptomatology (according to the PHQ-2 and GAD-2) of adults participating in the National Health Interview Survey (NHIS; January-June 2019) with adults participating in the Household Pulse Survey (HPS; April-May 2020). They found that American adults participating in the HPS were three to four times as likely to screen positive for anxiety or depression disorders, or screen positive for both in April–May 2020, compared to American adults who were assessed in the first half of 2019. During the pandemic about 30% screened positive for these disorders. Importantly, the NHIS participants were asked about symptoms in the last two weeks and HPS participants were asked about symptoms in the last seven days. The study by McGinty et al. [8] found that of the adult Americans who participated in the Johns Hopkins COVID-19 Civic Life and Public Health Survey (April 2020) 13.6% reported symptoms of serious psychological distress compared to 3.9% of the adults who participated in the National Health Interview Survey (NHIS) in 2018 according to the Kessler 6 scale. Daly et al. [9] compared the prevalence of depression symptoms according to the PHQ-2 among American adults participating in the National Health and Nutrition Examination Survey (NHNE) of 2017-2018, and among American adults who participated in the Understanding America Study (UAS) in March and April 2020. Results showed a significant higher prevalence in March (10.6%) and April (14.4%) 2020 UAS samples compared to the 2017-2018 (8.7%) NHNE sample. Pierce et al. [10] focused on UK residents of 16 years and older who participated in the UK Household Longitudinal Study and were asked to complete a COVID-19 web survey. Their findings showed that mean scores on the GHQ-12 increased significantly from 11.5 in 2018–2019 to 12.6 in April 2020. The increase was not simply a continuation of previous upwards trends from 2014 to 2019. The prevalence of clinically significant mental distress according to the GHQ-12, which should be distinguished from a mental disorder, among the total study sample increased from 18.9% in 2018–2019 to 27.3% in April 2020. The study by Van der

Velden et al. [11] focused on Dutch adults using the data of surveys conducted with the population-based longitudinal LISS panel. Contrary to the previous population-based studies, no increase was found in anxiety and depression symptoms according to the MHI-5 during the COVID-19 outbreak (March 2020) compared to pre-outbreak levels (November 2019). To account for possible seasonal effects, they also assessed the course of symptoms in the same period one year earlier (November 2018- March 2019), showing a similar pattern ruling out the possibility that findings can be attributed to a seasonal effect.

The differences in results between the studies in US and UK on the one side and in the Netherlands on the other side may partly be a result of (slightly) different study periods, besides differences in instruments, use of different samples to compare pre- and post-outbreak prevalence [7, 8, 9], governmental responses, and health care and social welfare systems. Perhaps the negative effects of the pandemic on the mental health of the general population are especially prevalent in the months after the COVID-19 outbreak. To examine a possible increase in mental health problems (anxiety and depression symptoms) after the COVID outbreak, the data of a new survey conducted in June 2020 was analyzed with the following first research question:

1. *To what extent did the post-outbreak prevalence of anxiety and depression symptom levels (June 2020) differ from the pre-outbreak (November 2019) and peri-outbreak (March 2020) prevalence?*

The sudden societal changes and related stressors after the COVID-19 outbreak may also have worsened symptoms or have hindered the recovery of symptoms among individuals with existing mental health problems. In addition, the pandemic may have reduced access to or use of mental

health care services, or forced mental health care workers to continue the treatment using online facilities instead of usual vis-à-vis contacts contributing to a deteriorating mental health [12, 13].

However, the review by Sheridan Rains et al. [13] revealed that most studies on this important topic documented observations and experiences rather than reporting on research based on empirical data. To the best of our knowledge, to date only one empirical study focused on this issue. Based on a cross-sectional case-control study conducted at the end of February 2020, Hao et al. [14] revealed that non-psychotic psychiatric patients had higher COVID-19 related PTSD symptoms, and higher anxiety, depression, and stress symptom levels than the healthy control group. However, it is unknown if differences in anxiety, depression, and stress symptom levels were larger than under 'normal' circumstances. No empirical study has been conducted comparing pre-outbreak recovery-rates with post-outbreak recovery rates among those with existing mental health problems. The second research question is therefore:

2. *To what extent did individuals with anxiety and depression symptom levels during the outbreak (March 2020) differ in recovery of symptoms about three months later (June 2020), from the recovery of individuals with pre-outbreak symptoms (November-December 2019) about three months later (March 2020)?*

Lockdowns and social distancing (including stay-at-home orders) are important preventive measures that may help to reduce the spread of COVID-19, but social isolation could lead to a rise of loneliness [15, 16] creating the so-called social connectivity paradox [15]. Non-COVID-19 studies have shown that loneliness, e.g. the subjective feeling referring to the perceived inadequacy of one's social relationships, is very prevalent and may be considered a major public concern. Loneliness is negatively associated with all-cause mortality, worse mental health and negative cardiovascular outcomes [17, 18, 19].

The limited number of COVID-19 related longitudinal studies on loneliness among the general population showed somewhat mixed results. In the earlier mentioned study by McGinty et al. [8], 13.8% always or often felt lonely in April 2020. Based on a comparison with another national survey conducted in April-May 2018, the authors concluded that loneliness increased only slightly (from 11% to 13.8%). Bu et al. [20] compared loneliness data of the large UK Household Longitudinal Study (wave 9; January 2017- June 2019) with loneliness data of the COVID-19 Social Study (March-May 2020), and found that loneliness levels were higher in the COVID-19 Social Study. In the longitudinal study by Killgore et al. [21], using the US Mechanical Turk (MTurk) crowdsourcing platform, loneliness increased significantly from March to June 2020, particularly from April to May 2020. Only one longitudinal study among the adult general population was identified using pre-outbreak data on loneliness of the study sample. Luchetti et al. [22] assessed trajectories of loneliness among a nationwide sample of American adults in response to COVID-19, using data of assessments in January/early February 2020 (before the outbreak), late March (during the President's initial "15 Days to Slow the Spread" campaign), and late April (during the "stay-at-home" policies of most states). In study no significant changes in loneliness were found among the total study sample, in contrast to an increase in social support.

In addition, current population wide studies treated loneliness as a unidimensional construct reflected by sum scores of items. The study by Hyland et al. [23] provided support for the presence of four exclusive subtypes of loneliness, e.g. a low loneliness, social loneliness, emotional loneliness, and social loneliness and emotional loneliness subtype. To gain further insight in the effects of this pandemic on loneliness, we therefore formulated the following third research question:

3. *To what extent did the post-outbreak prevalence of emotional loneliness, social loneliness, and social and emotional loneliness (June 2020) differ from the pre-outbreak prevalence (October 2019)?*

In line with the results of non-COVID-19 studies, COVID-19 related studies among the general adult population found positive relationships between loneliness and mental health problems. For example, the large cross-sectional study by González-Sanguino et al. [24] among a sample of Spanish adults found that loneliness was significantly correlated with depression, anxiety, and posttraumatic stress symptoms at the end of March 2020. The large cross-sectional study by Li and Wang [25], conducted at the end of April 2020 among adult UK residents, showed that loneliness was positively associated with having COVID-19 related symptoms. In the cross-sectional study by Palgi et al. [26], conducted between March 15 and April 2020, loneliness among a large sample of Israeli was positively related with depression and general anxiety symptoms. The study by Killgore et al. [21] among American adults showed that loneliness was significantly correlated with depression and suicidal ideation at the three time points during the pandemic.

The findings above suggest that individuals who were lonely before or during the pandemic more often suffered from mental health problems during this period (from pre-, peri- to post-outbreak) compared to individuals who were not lonely. However, it is unknown to what extent mental health problems increased (or decreased) among individuals who were lonely before *and* after the outbreak, among individuals who were lonely before *or* after the outbreak, and individuals who were not lonely in the pre-outbreak period. This generates the fourth and last research question:

4. *To what extent were individuals with pre-outbreak (October 2019) and post-outbreak (June 2020) emotional and/or social loneliness more at risk for pre- (November 2019), peri- (March 2020) and/or post-outbreak (June 2020) anxiety and depression symptoms?*

The objective of this prospective study is to answer these four above mentioned research questions using a Dutch population-based probability sample. Respondents participated in surveys in October 2019 (T1), November 2019 (T2), March 2020 (T3), and June 2020 (T4). With respect to anxiety and depression symptoms we focused on the prevalence of moderate-high anxiety and depression symptom levels, and with respect to recovery we made a distinction between the recovery-categories ‘remission’, ‘improved’, ‘unchanged’ and ‘worsened’. Concerning loneliness, we focused on the following subtypes of loneliness: ‘low loneliness’, ‘emotional loneliness’, ‘social loneliness’, and ‘social and emotional’ loneliness.

Materials and methods

Procedures and participants

We extracted and merged data from the Longitudinal Internet studies for the Social Sciences (LISS) panel. This panel is administered by CentERdata and the set-up was funded by the Dutch Research Council (NWO). The LISS panel is based on a traditional probability sample drawn from the Dutch population register by Statistics Netherlands [27]. Participants receive an incentive of 15 euros per hour. Panel members who do not have a computer and/or internet access are provided with the necessary equipment at home (further information about the LISS panel, conducted studies since 2007, and open access data see:

https://www.dataarchive.lissdata.nl/; in English). Data of the LISS panel (until March 2020) was also used in the COVID-19 study by van der Velden et al. [11].

Data on loneliness were extracted from two surveys: the (longitudinal) Social Integration and Leisure module of the LISS panel, wave October 2019 (T1: $N^{invited}$=5,929, response=84.2%), and the ‘Effects of the Outbreak of Covid-19’-survey conducted in June 2020 (T4: $N^{invited}$=7,022, response=79.7%). Data on anxiety and depression symptoms were extracted from three surveys: from the longitudinal Health module of the LISS panel, wave November 2019 (T2: $N^{invited}$=5,954, response=86.4%), the third wave of the Victims in Modern Society survey [11] survey conducted in March 2020 (T3: $N^{invited}$=6,568, response=83.6%), and the above mentioned ‘Effects of the Outbreak of Covid-19 survey’ conducted in June 2020 (T4). In total 4,084 respondents participated in all four surveys. To optimize the representativeness of the study sample, we weighted the data using 16 exclusive demographic profiles among the total adult Dutch population 2019 (N^{2019}=13,926,066), based on the data of Statistics Netherlands (see: https://opendata.cbs.nl/#/CBS/en/; in English). The 16 profiles were constructed using the following demographic characteristics at the end of 2019: sex (man, woman), age (18- 34, 35-49, 50-64, 65 years and older) and marital status (married and unmarried), yielding 2^*4^*2=16 exclusive demographic profiles. All results are based on the total weighted sample.

Ethical approval and informed consent

According to the Dutch Medical Research Involving Human Subjects Act (WMO) the present study did not require approval from a Medical Ethical Testing Committee (METC). Nevertheless, the longitudinal Social Integration and Leisure module and Health module (as part of the Longitudinal Core Study in LISS, starting in 2007) were evaluated and approved by the

Board of Overseers, an Internal Review Board (IRB) until 2014. The VICTIMS survey (starting in 2018) was approved by an Institutional Review Board, consisting of external and internal reviewers of CentERdata. The ‘Effects of the Outbreak of Covid-19’ survey was conducted by the Institute for Applied Microeconomics of Universität Bonn in Germany, and in Germany no medical ethical approval is required for this type of research. The ‘Effects of the Outbreak of COVID-19’ survey was evaluated and approved by internal reviewers of CentERdata not involved in designing this study, and used the same questions on mental health and loneliness as the surveys approved by the IRB. In accordance with the General Data Protection Regulation (GDPR), participants gave explicit consent for the use of the collected data for scientific and policy relevant research.

Measures

Anxiety and depression symptoms

Anxiety and depression symptoms were assessed at T2, T3 and T4 using the Mental Health Index or Inventory (5-item subscale of the MOS 36-item short-form health survey) [28, 29]. The MHI-5 asks respondents to rate their mental health during the past month on 6-point Likert scales, such as ‘This past month I felt very anxious’ and ‘I felt depressed and gloomy’ (0=never, 1=seldom, 2=sometimes, 3=often, 4=mostly, 5=continuously). After recoding the three negative formulated items, the total scores were computed and multiplied by four (to arrive at a 0-100 scale) where lower scores indicate more anxiety and depression symptoms levels (all Cronbach’s Alpha’s ≥ 0.87). A cut-off score of < 60 [30] was used to identify respondents with moderate to high anxiety and depression symptom levels.

Loneliness

Loneliness was assessed at T1 and T4, using the 6-item De Jong Gierveld Loneliness Scale [31, 32]. Respondents are asked to rate items such as ‘I often feel deserted’ and ‘there are enough people I can count on in case of a misfortune’ on three-point Likert scales (1=yes, 2=more or less, 3=no). According to the scoring guidelines the items were dichotomized to reflect the ‘presence’ or ‘absence’ of an indicator (item) of loneliness. For the emotional loneliness items, agreement responses were taken to indicate item endorsement, while for the social loneliness items, disagreement responses were taken to indicate item endorsement. Given the analytic strategy (see below) we did not compute Cronbach’s Alpha’s.

Physical illness

Physical illness was assessed at T2. The Health module assessed several Physician-diagnosed Diseases (PD) in the past year (1=yes, 2=no) and Health Problems (HP) respondents regularly suffer from (1=yes, 0=no). For the present study we focused on reported: 1.) lung problems ((PD=chronic lung disease such as chronic bronchitis or emphysema or asthma) or (HP=short of breath, problems with breathing, or coughing, a stuffy nose or flu-related complaints)); 2.) heart problems ((PD=angina, pain in the chest, a heart attack including infarction or coronary thrombosis or another heart problem including heart failure) or (HP=heart complaints or angina, pain in the chest due to exertion)); and 3.) diabetes (PD=diabetes or a too high blood sugar level).

Data analyses

Repeated multivariate logistic regression analyses were conducted to answer the first research question, while controlling for sex and pre-outbreak age, education level, employment status, domestic situation, employment status, ethnicity, lung problems, heart problems, and diabetes at the end of 2019 (November-December).

To answer the second research question, we applied the Reliable Change Index (RCI) and clinical cut-off (CO)-score [33] to gain insight in how many respondents with moderate-high symptom levels at T2 recovered (remission), improved, faced similar symptom levels (unchanged) or suffered from higher symptom levels at T3 (worsened). The psychometric data required to compute both RCI and CO was based on the data of the total weighted sample at T2 (general population; N=5.157). Computation was done as follows:

1.) $RCI=1.96^{*}\sqrt{}\ 2\ (SEM)^{2}$, with $SEM=SD^{*}(1\text{-Cronbach's }\alpha)$; and

2.) $CO=((M^{\text{mod.-high symptom subgroup}*}SD^{\text{general population}}) + (M^{\text{gen. population}*}SD^{\text{mod.high symptom subgroup}}))/$ $(SD^{\text{general }*}SD^{\text{mod.high symptom subgroup}})]$.

The prevalence of four categories of recovery (change) at T3 among respondents with moderate-high anxiety and depression scores at T2 were computed as follows [34]:

1.) *Remission* (($M^{\text{difference T2-T3}} \geq$ RCI) and ($M^{T2} \geq M^{T3}$) and ($M^{T3} <$ CO));

2.) *Improved* (($M^{\text{difference T2-T3}} \geq$ RCI) and ($M^{T2} \geq M^{T3}$) and ($M^{T3\,\geq}$ CO));

3.) *Unchanged* ($M^{\text{difference T2-T3}} <$ RCI); and

4.) *Worsened* (($M^{\text{difference T2-T3}} <$ RCI) and ($M^{T2} < M^{T3}$) nd ($M^{T3} \geq$ CO)).

The same procedure was used to assess recovery at T4 among those with moderate-high anxiety and depression symptom levels at T3. For these analyses MHI-scores were reversed (100-score), where higher scores reflect more symptoms.

The subgroup of respondents with moderate-high anxiety and depression symptom levels at T3 partly overlapped with the subgroup with high symptom levels at T2. Of the 961

respondents with high symptom levels at T2 *or* T3, 431 had high symptoms levels at T2 *and* T3. Because of this overlap, differences in the distribution of these four recovery categories at T3 and T4 could not directly be assessed using a chi-squared test. To solve this non-independence problem and to be able to compare the distribution of recovery categories at T3 and T4 we used the following analytic strategy to obtain exclusive subsamples. Firstly, we randomly split the total study sample into two almost equal groups of respondents (A and B), This resulted in two independent subgroups with high symptom levels at T2 (subgroup A1 and B1) and two independent subgroups with high symptoms levels at T3 (subgroup A1 and B2). Secondly, we assessed the differences in the distribution of recovery categories between the independent subgroups A1 and B2, and between the independent subgroups A2 and B1 using a chi-squared test.

To answer the third research question, we first performed latent class analyses (LCA) to identify subtypes of loneliness with the dichotomized items of the De Jong Gierveld Loneliness scale as indicators. The analyses were performed for T1 and T4 separately. Given the results of Hyland et al. [23] that identified four classes using the instrument, we limit the comparisons of one to seven classes by assessing the relative fit based on reductions in the Bayesian information criterion (BIC), the sample size adjusted BIC, as well as bivariate residuals (BVR’s) [35, 36]. The most parsimonious model with the lowest BIC and sample size adjusted BIC was chosen, while examining the corresponding BVR’s of the model that seemed to be the most parsimonious (BVR’s larger than 3.84 indicate that the estimated models failed to account for the pairwise association of the specific indicators or covariates). In addition, we looked at entropy values that are measures of the quality of classification (approaching one indicates clear delineation of classe) [37]. LCA was conducted using LatentGold 5.1. Subsequently, class membership was added to the respondents’ data. Possible changes in loneliness, e.g. changes in the prevalence of

identified classes, were assessed using repeated multivariate logistic regression while controlling for the same variables. All non-LCA analyses were conducted with IBM SPSS version 26.

For the fourth research question, multivariate logistic regression analyses and repeated multivariate logistic regression were performed with pre-, peri- and post-outbreak anxiety and depression symptoms as dependent variables, while controlling for the same variables as in the previous analyses. Similar to Hyland et al. [23] the LCA revealed four classes at T1 and T4. The four pre-outbreak (T1) and four post-outbreak (T4) classes were used to construct four exclusive subgroups consisting of respondents who were:

1.) *not lonely at T1 and not lonely at T4*;

2.) *not lonely at T1, but social and/or emotional lonely* at T4;

3.) *social and/or emotional lonely at T1, but not lonely at T4*; and

4.) *social and/or emotional lonely at both T1 and T4*.

Although 4*4 subgroups can be distinguished using the four loneliness classes at T1 and T4, we limited the number of subgroups because of low cell counts in several subgroups (see S1 Appendix), but also to improve the power of the statistical tests. Four repeated multivariate logistic regression analyses were performed to examine the course of moderate-high anxiety and depression symptom levels (T2 to T4) within each subgroup, while series of multivariate logistic regression were performed to examine differences between those four subgroups in the prevalence of moderate-high anxiety and depression symptom levels at T2, T3 and T4.

Results

Characteristics respondents

The characteristics of the respondents are shown in Table 1.

Table 1 Characteristics study sample (N=4,084)

	November - December 2019
	n (%)
Sex	
- man	2,012 (49.3)
- woman	2,072 (50.7)
Education level	
- low	991 (24.3)
- medium	1,474 (36.1)
- high	1,620 (39.7)
Domestic situation	
- (un)married cohabitation without child(ren)	1,399 (34.3)
- (un)married cohabitation with child(ren)	1,382 (33.8)
- single with child(ren)	237 (5.8)
- single	947 (23.2)
- other	118 (2.9)
Employment status	
- paid employment	2,005 (49.1)
- self-employed	223 (5.5)
- unemployed/job seeker	96 (2.4)
- student	345 (8.4)
- takes care of housekeeping	317 (7.8)
- pensioner	775 (19.0)
- has (partial) work disability	185 (4.5)
- other	138 (3.4)
Age categories (in years)	
- 18-34	1,089 (26.7)
- 35-49	966 (23.7)
- 50-64	1,057 (25.9)
- 65 and older	972 (23.8)
Ethnicity	
- native	3,203 (78.4)
- non-native	881 (21.6)
Lung problems	
- no	3,258 (79.8)
- yes	826 (20.2)
Heart problems	
- no	3,825 (93.7)
- yes	259 (6.3)

Diabetes	
- no	3,908 (95.7)
- yes	176 (4.3)

self-employed = autonomous professional, freelancer, self-employed, or works or assists in family business. Educational level: low = primary education, preparatory intermediate vocational education, or other, medium = higher general secondary/pre-university education, intermediate professional education, high = higher professional education/university.

Changes in prevalence anxiety and depression symptoms

The prevalence of moderate-high anxiety and depression symptom levels was 16.8%, 17.2% and 15.3% at T2, T3 and T4 respectively. Repeated multivariate logistic regression analyses showed that the prevalence decreased significantly at T4 compared to T2 (adjusted OR (AOR)=0.88, 95% CI=0.79-0.99, p=0.026), and compared to T3 (AOR=0.88, 95% CI=0.81-0.97, p=0.007), whereas it did not change significantly between T2 and T3 (AOR=1.02, 95% CI=0.94-1.12, p=0.611).

Recovery anxiety and depression symptoms in months after T2 and T3

In Table 2 the prevalence of the four recovery categories at T3 and T4 of the total study sample and the two split-half samples are presented. A Chi-squared test revealed no significant differences in the distribution of recovery categories at T3 and T4 between subgroup A1 and B2 (χ^2=3.726, df=3, p=0.293), nor between subgroup A2 and B1 (χ^2=2.58, df=3, p=0.461) respectively. These analyses were repeated among those with high anxiety and depression symptom levels (using cut-off < 45 of original scale). No differences in recovery were found (see S2 Appendix). Because of the random sampling to obtain the two half-split samples (see Method section), we repeated the random sampling to rule out the possibility that findings can be attributed to one specific sampling. These control analyses also showed no differences.

Table 2 Symptoms, recovery, loneliness before and during the COVID-19 among general population

	Anxiety and depression symptoms at T3/T4						
				split-half groups[1]			
	Total group			Subgroup A1		Subgroup B1	
	n	%		n	%	N	%
Anxiety and depression symptoms at T2							
- remission at T3	110	16.1		56	17.0	58	16.2
- improved at T3	37	5.4		16	4.9	17	4.8
- unchanged at T3	373	54.3		179	54.4	194	54.3
- worsened at T3	166	24.2		78	23.7	88	24.6
	Total group			Subgroup A2		Subgroup B2	
	n	%		n	%	n	%
Anxiety and depression symptoms at T3							
- remission at T4	127	18.0		69	20.2	57	15.7
- improved at T4	42	6.0		18	5.3	25	6.9
- unchanged at T4	395	56.1		183	53.5	213	58.5
- worsened at T4	140	19.9		72	21.1	69	19.0

[1]The total study sample (N=4084) was randomly split in two exclusive subgroups (subgroup A=2038 and subgroup B=2046). Due to weighting the distribution is not exactly 50-50% and therefore the numbers of the subgroups sometimes differ from the total numbers. For all four subgroups recovery was computed like the computations of recovery at T3 and T4 among the total group with moderate-high symptom levels at T2 and T3, using the M's and SD's of the four subgroups.

Loneliness subtypes

The results of the LCA showed, similar to Hyland et al. (2019), that the 4-class solution offered the best parsimonious fit with the lowest BIC and SABIC values, relatively low classification errors (≤ 5%) and high Entropy R’s (≥ 0.91) at both T1 and T4 (see S3 Appendix for all details). Given the probabilities of endorsing loneliness items on the four identified classes at T1 and T4 (see S4 Appendix for all details), in parallel with Hyland et al. (2019), class 1 was labelled “low loneliness”, class 2 “emotional loneliness”, class 3 “social and emotional loneliness” and class 4

was labelled “social loneliness”. The distribution of classes at T1 and T4 is presented in Table 3 and indicates that the large majority was not social and/or emotional lonely at T1 or T4. The prevalence of anxiety and depression symptoms at T2 among the low loneliness, emotional loneliness, social and emotional loneliness, and social loneliness class at T1 (one month before T2) differed significantly (χ^2=612, 29, df=3, p <.001). The prevalence was 8.9%, 40.5%, 54.2% and 25.4% respectively. The same pattern was found at T4 (χ^2=786.32, df=3, p <.001): the prevalence was 5.5%, 36.3%, 60.6%, and 23.9% respectively.

Table 3 Course of loneliness classes

	October 2019 (T1)		June 2020 (T4)		
Loneliness	n	%	n	%	AOR (95% CI)
- low loneliness (class 1)	3,095	75.8	2,862	70.1	0.68 (0.63-0.74)***
- emotional loneliness (class 2)	753	18.4	1014	24.8	1.61 (1.48-1.76)***
- social and emotional loneliness (class 3)	168	4.1	142	3.5	0.84 (0.69-1.01)
- social loneliness (class 4)	68	1.7	67	1.6	0.97 (0.72-1.30)[1]

AOR=Odd Ratios adjusted for age, sex, education, employment status, domestic situation, employment status, ethnicity, lung problems, heart problems, diabetes. 95% CI=95% Confidence interval. Due to weighting, numbers may slightly differ between Tables. *** p <.001.
[1] AOR without employment status as covariate because of computation problems due to low cell counts.

Changes in loneliness

Repeated multivariate logistic regression analyses (see Table 3) showed that the prevalence of respondents with low loneliness decreased significantly between T1 and T4 (75.8% and 70.1% respectively), while the prevalence of emotional loneliness increased in this period (18.4% and 24.4% respectively). The prevalence of ‘social and emotional loneliness’ and ‘social loneliness’ did not differ significantly between T1 and T4.

The associations between changes in loneliness, and anxiety and depression symptoms

Table 4 provides an overview of the prevalence of moderate-high anxiety and depression symptom levels at T2, T3 and T4 of the subgroups with and without changes in loneliness [$N^{\text{not lonely at T1, not lonely at T4}}$=2559 (62.7%); $N^{\text{not lonely at T1, lonely at T4}}$=536 (13.1%); $N^{\text{lonely at T1, not lonely T4}}$=303 (7.4%); $N^{\text{lonely at T1, lonely at T4}}$=686 (16.8%)]. The results of the repeated multivariate logistic regression analyses show no changes in the prevalence of anxiety and depression symptoms between T2 and T3 across the four subgroups. However, in the ‘not lonely at T1, not lonely T4’ subgroup’ and ‘lonely at T1, not lonely at T4’ subgroup, the prevalence of anxiety and depression symptoms decreased significantly to 4.4% and 14.5% respectively. In subgroup ‘not lonely at T1, lonely at T4’ the prevalence rose significantly from 17.9% at T1 to 26.3% at T4.

Despite the absence of changes in the prevalence of anxiety and depression symptoms between T2 and T3 across the four subgroups based on the loneliness classes at T1 and T4, the multivariate logistic regression clearly show significant differences in the prevalence between the four groups at T2, T3, and T4. For example, at T4, the prevalence of moderate-high anxiety and depression symptoms levels differed significantly between all subgroups (4.4%, 26.3%, 14.5% and 48% respectively). As expected, respondents who were not lonely at pre- and post-outbreak had the lowest prevalence of moderate-high anxiety and depression symptom levels at T2, T3 and T4.

Table 4 Course of loneliness and anxiety and depression symptoms

	Anxiety and depression symptoms								
	November 2019 (T2)		March 2020 (T3)		June 2020 (T4)		T2 vs. T3	T2 vs. T4	T3 vs. T4
Loneliness	n	%	n	%	n	%	AOR (95% CI)	AOR (95% CI)	AOR (95% CI)
- not lonely at T1, not lonely at T4	178	7.0[a]	205	8.0[a]	112	4.4[a]	1.17 (0.99-1.39)	0.62 (0.49-0.77)***	0.52 (0.43-0.64)***
- not lonely at T1, lonely at T4	96	17.9[b]	104	19.4[b]	141	26.3[b]	1.10 (0.86-1.39)	1.63 (1.24-2.14)***	1.49 (1.18-1.87)***
- lonely at T1, not lonely T4	65	21.5[b]	64	21.1[b]	44	14.5[c]	0.91 (0.67-1.24)	0.60 (0.41-0.90)*	0.66 (0.47-0.92)*
- lonely at T1, lonely at T4	348	50.7[c]	332	48.4[c]	329	48.0[d]	0.88 (0.75-1.04)	0.88 (0.72-1.07)	0.99 (0.84-1.17)

AOR=Odd Ratios adjusted for age, sex, education, employment status, domestic situation, employment status, ethnicity, lung problems, heart problems, diabetes. Percentages in a column with the same letter in superscript ([a, b, c, d]) do not differ significantly according to adjusted Odd Ratios. 95% CI=95% Confidence interval. Due to weighting, numbers may slightly differ between Tables. * $p < 0.05$, ** $p < 0.01$, *** $p < .001$. Lonely=social loneliness, emotional loneliness, or social and emotional loneliness.

Discussion

To date, the present study on the effects of COVID-19 on mental health and loneliness among the general population is one of the very few prospective studies based on probability-based samples of the general population, , using data on mental health and loneliness of the same study sample just before the COVID-19 outbreak (October-November 2019). The results showed the following clear patterns. Compared to the pre-outbreak (T2) and outbreak (T3) prevalence of anxiety and depression symptoms, the prevalence of post-outbreak (T4) symptoms did not increase but decreased slightly but significantly. These results seem to differ strongly from the studies by McGinty et al. [22] and Daly et al. [9], and especially Twenge and Joiner [7], but we cannot rule out the possibility that differences in study designs, among other things, play a role in these differences. For instance, these studies compared different pre- and post-COVID outbreak study samples instead of pre- and post-COVID-19 outbreak assessments within the same sample. However, the study by Pierce et al. [10], similar to our study, prospectively assessed mental distress among the same sample showing an increase from 2018-2019 to April 2020. On the other hand, in their population-based study Hyland et al. [38] concluded that the rates of screening positive for generalized anxiety disorder and depression in Ireland around the beginning of April 2020, did not differ markedly from those reported in previous non-COVID-19 national prevalence studies in the UK.

As described earlier [11] the absence of differences in anxiety and depression symptoms within the Dutch study sample may also be related to how the Dutch governments reacted and intervened, and related to the Dutch social welfare and health care systems in the Netherlands. Adult people without a job (or lost a job due to this pandemic) can invoke for unemployment

benefits and, in principle, each Dutch citizen has a health care insurance regardless of being employed or not. During the study period the Dutch government very quickly implemented financial support to companies who lost revenues significantly because of the COVID-19 pandemic to allow them to keep people at work. The governmental acts, interventions and management of this pandemic were, until now, not politized in the Netherlands, meaning that the compensation for lost salaries and profits was rather generous, preventing potential sources of additional financial worries and stress to the people concerned.

A relevant research topic not covered in previous longitudinal COVID-19 studies is the recovery of existing mental health problems during this pandemic [13]. We examined, using the Reliable Change Index (RCI) of Jacobson and Truax [33] the extent to which individuals with anxiety and depression symptoms at the beginning of the COVID-19 outbreak (T3) recovered a few months later (T4). We compared the distribution of the four recovery-categories (remission, improved, unchanged and worsened) with the distribution of these categories during the outbreak (T3) among those with anxiety and depression symptoms before the outbreak (T2) and found no significant differences. Moreover, additional analyses among individuals with very high existing MHI-5 scores showed similar results. In other words, we found no support in the data that the COVID-19 pandemic hindered recovery of anxiety and depression symptoms during the first four months. This suggest that we have to be careful to attribute worsening symptoms solely to this pandemic. In addition, our findings do not deny or neglect that people with existing anxiety and depression symptoms may have concerns about their mental illness worsening because of the COVID-19 pandemic [39].

In line with the study of Hyland et al. [23], the latent class analyses revealed four exclusive classes of loneliness: the low loneliness, emotional loneliness, social loneliness, and emotional and social loneliness class. Findings with respect to the course of loneliness duting the

pandemic differed partly from the findings with respect to anxiety and depression symptoms. The analyses showed that the prevalence of emotional loneliness increased after the outbreak (18.4% versus 24.8%), while the prevalence of individuals who were social lonely or social and emotional lonely did not differ from the pre-outbreak prevalence. The prevalence of individuals with low loneliness decreased slightly from 75.8% to 70.1%, suggesting that the lockdowns and social distancing (including stay-at-home orders) negatively affected loneliness for a relatively small group [8, 10, 21]. The absence of differences between pre- and post-outbreak social loneliness and social and emotional loneliness suggest that people have found ways to keep social contacts, such as via (mobile) telephone, social media (e.g. Whatsapp, Facebook) and video calls (e.g. Skype, ZOOM), but that these communication facilities not always provide emotional connectedness. Although we controlled for demographics and physical illness, it was outside the aim and scope of the present study to further discuss which groups were more at risk to become emotional lonely. Our results seem to differ from the outcomes of the prospective study of Luchetti et al. [22], the only other prospective study on loneliness among the general population using a longitudinal sample. In their study, however, loneliness was treated as a unidimensional concept.

As expected, and in line with previous research, individuals with persistent loneliness (lonely before and after the COVID-19 outbreak) much more often suffered from anxiety and depression symptoms before, during and after the outbreak (prevalence ≥ 48%) than individuals who were not lonely before and after the outbreak (prevalence ≤ 8%). Importantly, results furthermore showed that the prevalence of symptoms among the four subgroups (not lonely at T1, not lonely at T4; not lonely at T1, lonely at T4; lonely at T1, not lonely at T4; lonely at T1, lonely at T4) did not change between November 2019 and March 2020 suggesting that the effects of all social distancing measures took place after the outbreak. Interestingly, among those who

became lonely, the prevalence of symptoms increased, while among those who were not lonely anymore after the outbreak or not lonely before and after the outbreak, the prevalence of symptoms decreased after the outbreak (June 2020). These findings indicate that loneliness plays a key role in the presence of anxiety and depression symptoms during this pandemic. The results also suggest that COVID-19 related mental health policies should target individuals who became lonely after the COVID-19 outbreak (13.1% of the total study sample) and especially target individuals who were and are lonely after the outbreak (16.8% of the total study sample). Unfortunately, due to the low cell counts we could not examine the prevalence of symptoms among all sixteen combinations of the pre- and post-outbreak loneliness classes. Future studies are warranted to examine loneliness on the longer term to assess the extent to which patterns in loneliness remain stable or change.

Strengths and limitations

This study has its strengths and limitations. The prospective study design with pre-COVID-19 data on loneliness, anxiety and depression symptoms, use of a longitudinal population-based probability sample with high response-rates, and use of well-validated questionnaires are major strengths of the current study. We used the data of a large group of respondents (N=4,084) who participated in all four waves. However, we did not conduct clinical interviews to assess mental disorders such as generalized anxiety disorder and schizophrenia nor examined other relevant mental health problems such as sleeping problems and fatigue, which would have enriched our study. For instance, we cannot rule out the possibility that the recovery of depression, fatigue or sleeping problems after the COVID-19 outbreak is, due to all (practical) changes, lower than before the outbreak. In addition, we were not able to assess mental health consequences among

people specific mental disorders such as schizophrenia [40]. The most recent assessment in this study took place in June 2020 and future follow-ups are needed to gain insight in the effects of the pandemic on the longer term given the uncertainties about how this pandemic will develop and when effective vaccines will become available. In addition, we did not include children and adolescents of the Dutch general population who might be affected differently by the COVID-19 pandemic. We have no data on anxiety and depression symptoms, and loneliness covering an exact similar study period (October-November to June) to rule out possible seasonal effects: it is basically possible that for instance the decrease of the prevalence of anxiety and depression symptoms is caused by this effect. Interestingly, the study by Wang et al. [41] showed a significant reduction in depression and anxiety symptoms during the stressful first four weeks after the COVID-19 outbreak in China.

Final conclusions

Taken together, these results suggest that the Dutch population was capable to cope with and to adjust successfully to the drastic changes of their lives as a result of the COVID-19 pandemic outbreak during the first four months of 2020 (March 2020 to June 2020; cf. [11, 42, 43]. We did not find an increase in the prevalence of anxiety and depression symptoms during the first four months of the outbreak. And among those with symptoms we found similar recovery-rates during the first four months compared to before the pandemic. Nevertheless, the prevalence of emotional loneliness increased to some extent, and an increase in loneliness, especially persistent loneliness, was very strongly associated with the presence of anxiety and depression symptoms.

References

1. Rosa S de, Spaccarotella C, Basso C, Calabrò MP, Curcio A, Perrone Filardi P, Mancone M, Mercuro G, Muscoli S, Nodari S, Pedrinelli R, Sinagra G, Indolfie C. Reduction of hospitalizations for myocardial infarction in Italy in the COVID-19 era. Eur Heart J. 2020; ehaa409. https://doi.org/10.1093/eurheartj/ehaa409

2. Sheridan A, Andersen AL, Hansen ET, Johannesen N. Social distancing laws cause only small losses of economic activity during the COVID-19 pandemic in Scandinavia PNAS. 2020; 117: 20468-20473. https://doi.org/10.1073/pnas.2010068117

3. IMF. The Great Lockdown: Worst Economic Downturn Since the Great Depression. International Monetary Fund, 2020. https://blogs.imf.org/2020/04/14/the-great- lockdown-worst-economic-downturn-since-the-great-depression/ (accessed April 14, 2020).

4. IMF. COVID-19 Crisis Poses Threat to Financial Stability. International Monetary Fund, 2020. https://blogs.imf.org/2020/04/14/covid-19-crisis-poses-threat-to-financial- stability/ (accessed April 14, 2020).

5. Vindegaard N, Benros ME. COVID-19 pandemic and mental health consequences: Systematic review of the current evidence. Brain Behav Immun. 2020; S0889-1591(20)30954-5. https://doi.org/10.1016/j.bbi.2020.05.048

6. Xiong J, Lipsitz O, Nasri F, Lui LMW, Gill H, Phan L, Chen-Li D, Iacobucci M, Ho R, Majeed A, McIntyre RS, Impact of COVID-19 pandemic on mental health in the general population: A systematic review. J Affect Disord. 2020; 277: 55-64. https://doi.org/10.1016/j.jad.2020.08.001

7. Twenge JM, Joiner TE. U.S. Census Bureau- assessed prevalence of anxiety and depressive symptoms in 2019 and during the 2020 COVID- 19 pandemic. Depress. Anxiety 2020; 1-3. https://doi.org/10.1002/da.23077

8. McGinty EE, Presskreischer R, Han H, Barry CL. Psychological distress and loneliness reported by US adults in 2018 and April 2020. JAMA. 2020; 324: 93-94. https://doi.org/10.1001/jama.2020.9740

9. Daly M, Sutin A, Robinson E. Depression reported by US adults in 2017-2018 and March and April 2020. J Affect Disord. 2020. In press. https://doi.org/10.1016/j.jad.2020.09.065

10. Pierce M, Hope H, Ford T, Hatch S, Hotopf M, John A, Kontopantelis E, Webb R, Wessely S, McManus S, Abel KM. Mental health before and during the COVID-19 pandemic: a longitudinal probability sample survey of the UK population. Lancet Psychiatry. 2020; 21: S2215-0366(20)30308-4. https://doi.org/10.1016/S2215-0366(20)30308-4

11. Velden PG van der, Contino C, Das M, Loon P van, Bosmans MWG. Anxiety and depression symptoms, and lack of emotional support among the general population before and during the

COVID-19 pandemic. A prospective national study on prevalence and risk factors. J Affective Disord. 2020a. https://doi.org/10.1016/j.jad.2020.08.026

12. Schlegl S, Maier J, Meule A, Voderholzer U, 2020. Eating disorders in times of the COVID-19 pandemic-Results from an online survey of patients with anorexia nervosa. Int J Eat Disord. 2020; 1-10. https://doi.org/10.1002/eat.23374

13. Sheridan Rains L, Johnson S, Barnett P, Steare T, Needle JJ, Carr S, Lever Taylor B, Bentivegna, F, Edbrooke-Childs J, Scott HR, Rees J, Shah P, Lomani J, Chipp B, Barber N, Dedat Z, Oram S, Morant N, Simpson A, COVID-19 Mental Health Policy Research Unit Group, 2020. Early impacts of the COVID-19 pandemic on mental health care and on people with mental health conditions: framework synthesis of international experiences and responses. Soc Psychiatry Psychiatr Epidemiol. 2020; 1-12. https://doi.org/10.1007/s00127-020-1924-7

14. Hao F, Tan W, Jiang L, Zhang L, Zhao X, Zou Y, Hu Y, Luo X, Jiang X, McIntyre RS, Tran B, Sun J, Zhang Z, Ho R, Ho C, Tam W. Do psychiatric patients experience more psychiatric symptoms during COVID-19 pandemic and lockdown? A case-control study with service and research implications for immunopsychiatry. Brain Behav Immun. 2020; 87: 100-106. https://doi.org/10.1016/j.bbi.2020.04.069

15. Smith BJ, Lim MH, 2020. How the COVID-19 pandemic is focusing attention on loneliness and social isolation. Public Health Res Pract. 2020; 30; 3022008. https://doi.org/10.17061/phrp3022008

16. Philip KEJ, Polkey MI, Hopkinson NS, Steptoe A, Fancourt D. Social isolation, loneliness and physical performance in older-adults: fixed effects analyses of a cohort study. Sci Rep. 2020; 10: 13908. https://doi.org/10.1038/s41598-020-70483-3

17. Hawkley LC, Cacioppo JT. Loneliness matters: a theoretical and empirical review of consequences and mechanisms. Ann Behav Med. 2020; 40: 218–227. https://doi.org/10.1007/s12160-010-9210-8

18. Heinrich LM, Gullone E. The clinical significance of loneliness: a literature review. Clin Psychol Rev. 2006; 26: 695-718. https://doi.org/10.1016/j.cpr.2006.04.002

19. Leigh-Hunt N, Bagguley D, Bash K, Turner V, Turnbull S, Valtorta N, Caan C. An overview of systematic reviews on the public health consequences of social isolation and loneliness. Public Health, 2017; 152: 157-171. https://doi.org/10.1016/j.puhe.2017.07.035

20. Bu F, Steptoe A, Fancourt D. Who is lonely in lockdown? Cross-cohort analyses of predictors of loneliness before and during the COVID-19 pandemic. Public Health. 2020; 5: 31-34. https://doi.org/10.1016/j.puhe.2020.06.036

21. Killgore WDS, Cloonan SA, Taylor EC, Miller MA, Dailey NS. Three months of loneliness during the COVID-19 lockdown. Psychiatry Res. 2020; 293: 113392. https://doi.org/10.1016/j.psychres.2020.113392

22. Luchetti M, Lee JH, Aschwanden D, Sesker A, Strickhouser JE, Terracciano A, Sutin AR. The trajectory of loneliness in response to COVID-19. Am. Psychol. 2020. https://doi.org/10.1037/amp0000690

23. Hyland P, Shevlin M, Cloitre M, Karatzias T, Vallières F, McGinty G, Fox R, Power JM. Quality not quantity: loneliness subtypes, psychological trauma, and mental health in the US adult population. Soc Psychiatry Psychiatr Epidemiol. 2019; 5: 1089-1099. https://doi.org/10.1007/s00127-018-1597-8

24. González-Sanguino C, Ausín B, Castellanos MÁ, Saiz J, López-Gómez A, Ugidos C, Muñoz M Mental health consequences during the initial stage of the 2020 Coronavirus pandemic (COVID-19) in Spain. Brain Behav Immun. 2020; 87: 172-176. https://doi.org/10.1016/j.bbi.2020.05.040

25. Li LZ, Wang S. Prevalence and predictors of general psychiatric disorders and loneliness during COVID-19 in the United Kingdom. Psychiatry Res. 2020; 291: 113267. https://doi.org/10.1016/j.psychres.2020.113267

26. Palgi Y, Shrira A, Ring L, Bodner E, Avidor S, Bergman Y, Cohen-Fridel S, Keisari S, Hoffman YJ. The loneliness pandemic: Loneliness and other concomitants of depression, anxiety and their comorbidity during the COVID-19 outbreak. J Affect Disord. 2020; 275: 109-111. https://doi.org/10.1016/j.jad.2020.06.036

27. Scherpenzeel A, Das M. True longitudinal and probability based internet panels: evidence from The Netherlands. In: Das M, Ester P, Kaczmirek L, editors. Social and behavioral research and the internet: advances in applied methods and research strategies. Taylor & Francis, New York, 2011, 77–104. https://doi.org/10.4324/9780203844922

28. Ware JE, Sherbourne, CD. The MOS 36-item short-form health survey, SF-36: I. Conceptual framework and item selection. Med. Care 1992; 30; 473–483. https://doi.org/10.2307/3765916

29. Means-Christensen AJ, Arnau RC, Tonidandel AM, Bramson R, Meagher MW. An efficient method of identifying major depression and panic disorder in primary care. J Behav Med. 2005; 28: 565-572. https://doi.org/10.1007/s10865-005-9023-6

30. Driessen M. Een beschrijving van de MHI-5 in de gezondheidsmodule van het Permanent Onderzoek Leefsituatie [A Description of the MHI-5 in the Health Module of Permanent Research of Living Conditions, POLS]. Den Haag, Statistics Netherlands, 2011.

31. Jong Gierveld J de, Tilburg TG van. A 6-item scale for overall, emotional, and social loneliness: confirmatory tests on survey data. Res Aging. 2006; 28: 582-598. https://doi.org/10.1177/0164027506289723

32. Jong Gierveld J de, Tilburg T van. The De Jong Gierveld short scales for emotional and social loneliness: tested on data from 7 countries in the UN generations and gender surveys. Eur J Ageing. 2010; 7: 121-130. https://doi.org/10.1007/s1043 3-010-0144-6

33. Jacobson NS, Truax P. Clinical significance: A statistical approach to defining meaningful change in psychotherapy research. J Consult Clin Psychol. 1991; 59: 12–19. https://doi.org/10.1037//0022-006x.59.1.12

34. Dingemans AE, Son GE van, Aardoom JJ, Bruidegom K, Slof-Op 't Landt MC, Furth EF van. Predictors of psychological outcome in patients with eating disorders: A routine outcome monitoring study. Int J Eat Disord. 2016; 49: 863-873. https://doi.org/10.1002/eat.22560

35. Magidson J, Vermunt JK. Latent class models. In D. Kaplan (Ed.), Handbook of quantitative methodology for the social sciences (pp. 175–198). Thousand Oaks, CA: Sage Publications, 2004.

36. Vermunt, J.K, Magidson J. Latent GOLD 5.0 Upgrade Manual. Belmont MA, Statistical Innovations Inc, 2013.

37. Celeux G, Soromenho G. An entropy criterion for assessing the number of clusters in a mixture model. J. Classification 1996; 13: 195-212. https://doi.org/10.1007/BF01246098

38. Hyland P, Shevlin M, McBride O, Murphy J, Karatzias T, Bentall RP, Martinez A, Vallières F. Anxiety and depression in the Republic of Ireland during the COVID-19 pandemic. Acta Psychiatr Scand. 2020. In press. https://doi.org/10.1111/acps.13219

39. Costa M, Pavlo A, Reis G, Ponte K, Davidson L. COVID-19 Concerns Among Persons With Mental Illness. Psychiatr Serv. 2020; In press. https://doi.org/10.1176/appi.ps.202000245

40. Kozloff N, Mulsant BH, Stergiopoulos V, Voineskos AN. The COVID-19 global pandemic: implications for people with schizophrenia and related disorders. Schizophr Bull. 2020; 46: 752-757. https://doi.org/10.1093/schbul/sbaa051Advance

41. Wang C, Pan R, Wan X, Tan Y, Xu L, McIntyre RS, Choo FN, Tran B, Ho R, Sharma VK, Ho, C. A longitudinal study on the mental health of general population during the COVID-19 epidemic in China. Brain Behav Immun. 2020; 87: 40-48. https://doi.org/10.1016/j.bbi.2020.04.028

42. Chen S, Bonanno GA. Psychological adjustment during the global outbreak of COVID-19: A resilience perspective. Psychol Trauma. 2020; 12: s51–s54. https://doi.org/10.1037/tra000068510.1037/tra0000685

43. Wessely S. Don't panic! Short and long term psychological reactions to the new terrorism: the role of information and the authorities. J Mental Health. 2005; 14: 1-6. https://doi.org/10.1080/09638230500048099

Click here to access/download
Supporting Information
S1 appendix.docx

Click here to access/download
Supporting Information
S2 appendix.docx

Click here to access/download
Supporting Information
S3 appendix.docx

Click here to access/download
Supporting Information
S4 appendix.docx
